# Treatment of Xylazine-Associated Injection Skin Injuries

**DOI:** 10.1155/2024/8618440

**Published:** 2024-11-14

**Authors:** Ho-Man Yeung, William Mills Worrilow

**Affiliations:** Department of Medicine, Lewis Katz School of Medicine at Temple University, Philadelphia, Pennsylvania, USA

**Keywords:** skin injury, tranq wounds, wound care, xylazine

## Abstract

With the introduction of fentanyl and xylazine in the drug supply market, injection-related skin injuries and wounds are becoming more common. Xylazine is an alpha-2 adrenergic agonist thought to cause deep ulcerative wounds due to peripheral vasoconstriction leading to poor wound healing. This case series describes four patients with injection drug use leading to severe xylazine-related skin injuries who were treated between 2022 and 2023. This paper provides visualization of the extent and severity of these “tranq wounds,” as well as the healing progression when receiving medical treatment, addiction treatment, and wound care. Medical treatment and overall care were complicated by individual social determinants of health. Further understanding of xylazine-related wounds is necessary as xylazine continued to be an emerging threat in the United States. Though some reports in the literature capture the appearances, only few displayed progressive improvements or success in treatment given the challenging nature of treating this high-risk population.

## 1. Introduction

Xylazine is an alpha-2-adrenergic agonist commonly used as a veterinary anesthetic and sedative [1]. However, since 2016, xylazine (also colloquially known as “tranq” or “zombie drug”) has consistently been incorporated into the unregulated manufactured fentanyl supply due to its low cost and availability. When used together, xylazine can enhance the psychoactive effects of fentanyl [2]. Xylazine is considered an emerging threat, and its effect in humans is not fully known. Detrimental health concerns in persons who inject drugs (PWID) include overdose and wound-related infection. The exact mechanism for xylazine-associated skin injury is unclear, but it has been thought to be due to peripheral vasoconstriction leading to vascular damage. Other postulated causes of skin injury include cytotoxic effects, high frequency of injection, compression effects from prolonged sedation, and small vessel vasculopathy [2]. These wounds can occur at injection sites and noninjection sites of the skin. Wound infection is common due to poor living conditions, minimal access to health care, and inadequate hygiene.

Xylazine-associated skin injuries can be severe and difficult to treat, particularly if patients continue injection behaviors. These wounds often lead to skin and soft tissue infections and bacteremia, particularly *Staphylococcus aureus* and *Streptococcus pyogenes* [3, 4]. Our wound care clinic has been in operation to serve patients in low socioeconomic communities and communities of color. This is significant as nearly all unregulated fentanyl samples in our city contain xylazine [5].

Here, we report a case series of four patients with injection drug use and xylazine-associated wound. Though some reports in the literature capture the appearances, only few displayed progressive improvements or success in treatment given the challenging nature of treating this high-risk population. We believe sharing our clinical experience would further generate discussions and show that these wounds are indeed severe but can ultimately heal over time with treatment.

## 2. Case 1 Summary

**Figure 1:**
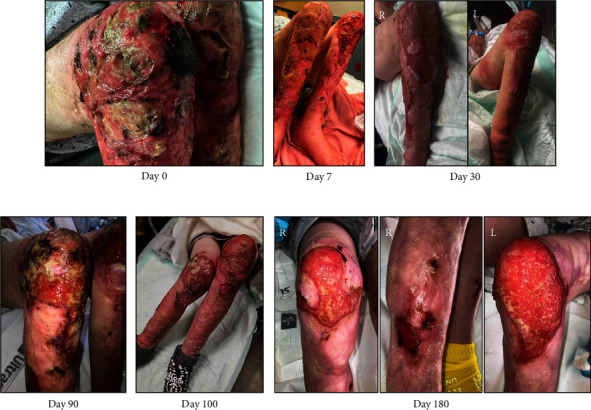
Bilateral lower extremity xylazine wounds.

A 28-year-old female, experiencing homelessness, with severe opioid dependence, injection drug use of 48–72 bags fentanyl/xylazine daily, chronic bilateral lower extremity wounds, and prior tibial and ischial osteomyelitis presented with worsening pain and drainage of her chronic leg wounds. Physical examination showed multiple eschar wounds on bilateral lower extremities with purulent drainage (Figure 1; Day 0). Wound care and burn surgery teams were consulted. Throughout her hospital course, the wound care team performed daily cleansing with saline and CarraKlenz, applied bacitracin to yellow/black tissue, applied Xeroform to all open areas, and dressed wounds with ABD pads, rolled gauze, and ACE wrap. Burn surgery performed wound debridement and biodegradable temporizing matrix (BTM) dermal substitute placement on her bilateral lower extremity wounds. After discharge from the hospital, she returned to use resulting in dermal substitute failure (Day 90). During the next six months, she had seven other admissions and would only get proper wound care during acute hospitalizations. Her wounds slowly improved, and she has been on methadone maintenance therapy. She remained homeless.

## 3. Case 2 Summary

**Figure 2:**
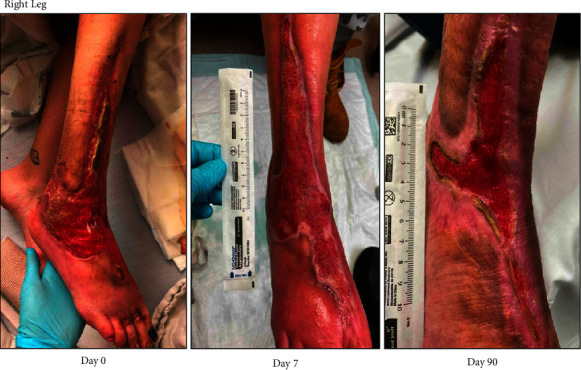
Right foot ulcerative wound with tendon exposure.

A 32-year-old female with opioid use disorder (4-5 bags fentanyl/xylazine daily) and chronic right lower extremity wounds previously complicated by tendonitis and cellulitis presented with tissue swelling and purulent drainage of the right foot and left wrist following fentanyl/xylazine use (Figure 2; Day 0). Three months prior, she underwent incision and drainage and fasciotomy. Since then, the patient reported exposed right foot extensor tendons with intermittent drainage. She was treated with clindamycin and doxycycline following growth of group A *Streptococcus* (GAS) and MRSA in aspirate cultures. After discharge, the patient followed closely with our outpatient wound care clinic three times weekly for xylazine-associated wound assessments. Her wound was cleansed with soap and water, topical application of bacitracin, and dressings with Kerlix and ACE bandages. Following discharge (Day 7), she continued with outpatient wound, with significant improvement, no signs of infection, and with granular tissue in wound bed. During the next five months, she was followed by our addiction medicine and internal medicine clinics and without any readmissions. She remained on buprenorphine treatment.

## 4. Case 3 Summary

**Figure 3:**
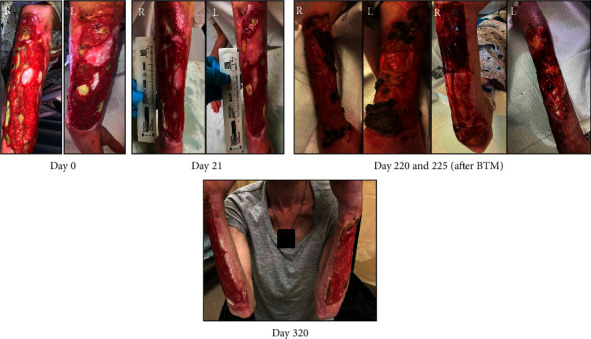
Bilateral upper extremity xylazine wound, treated with biodegradable temporizing matrix (BTM) dermal substitute.

A 40-year-old female with injection drug use of fentanyl/xylazine and methamphetamine presented with bilateral forearm wounds (Figure 3; Day 0). Over the past year, her forearm wounds had progressively worsened. She reported injecting 4–6 bags of fentanyl/xylazine into both arm wounds. On evaluation, there were no concerning signs for acute infection, though X-rays showed periosteal reaction at the right ulna suggesting chronic osteomyelitis. Given tendon exposure, there was concern for reinfection, so it was decided to hold antibiotic treatment for osteomyelitis until further wound healing with possible skin graft by plastic surgery. Blood cultures showed no growth. Throughout the patient's 14-day hospital stay, the wound care team applied silver sulfadiazine to her wounds and changed her dressing daily. She was discharged on buprenorphine–naloxone 8-2 mg three times daily. She followed up with our outpatient wound clinic with marked improvement in forearm wounds (Day 21). The patient was taught how to perform dressing changes daily and was given the supplies to do so. The wounds were treated with chlorhexidine gluconate wipes and dressed in silver sulfadiazine, Xeroform, ABD pads, and ACE wraps. The patient remained on buprenorphine without injection drug use until three months after discharge during which she began injecting 15 bags of fentanyl per day into her wounds and was readmitted to our hospital seven months after initial presentation (Day 220). She underwent BTM dermal substitute, and she continues to follow up with wound care and burn surgery clinic regularly while on buprenorphine treatment (Day 320).

## 5. Case 4 Summary

**Figure 4:**
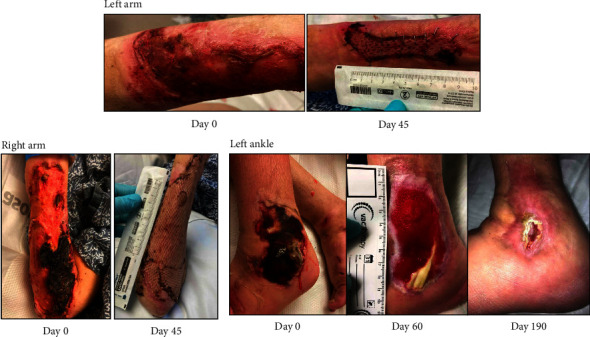
Multiple skin wounds of different extremities. Left ankle wound with tendon exposure.

A 33-year-old female with a history of opioid use disorder and injection behaviors of 70–80 bags of fentanyl/xylazine per day presented with painful, malodorous bilateral upper extremity wounds and left ankle wound (Figure 4; Day 0). The patient reported these wounds had worsened for the past six months. She is homeless and lives in her car. Blood cultures revealed MRSA and GAS bacteremia. She underwent debridement of her wounds followed by placement of two split thickness skin grafts on her arms bilaterally, with her thigh as donor site. While inpatient, negative pressure wound therapy was applied to her left ankle wound. She successfully completed a 6-week course of ceftriaxone and daptomycin and was maintained on buprenorphine therapy. Following her 60-day hospitalization, she was discharged to a medical respite with follow-up to our wound care clinic three times per week, who continued to exchange the patient's vacuum dressing at each visit. Petroleum gauze was applied over the exposed tendon followed by black foam at negative pressure 125 mmHg at each visit. After 190 days since her initial presentation, her left ankle wound had granulation tissue completely covering the previously exposed tendon and marked decrease in wound size. After her recovery, she was discharged from the medical respite.

## 6. Discussion

We presented four patients with significant social barriers combined with injection behaviors and severe wounds. Our main motivation to report our cases is to provide balance to what has been reported in the literature. Many reported cases or images available across the literature only display wounds upon presentation or initial evaluation [3, 4, 6–8]. Only a few demonstrated progression and healing of these wounds [9–11]. Since these images are publicly available and reported by the media, patients and clinicians may have false beliefs that these wounds do not heal or think that wound healing is strictly contingent upon substance recovery. Although injection behaviors and substance use are directly contributory, participation with community wound clinic and hospital treatment of wounds are greatly beneficial and reduce harm/infection risk in this vulnerable patient population. Despite the severity and extent of these wounds, there is healing potential with frequent wound care, in both the acute setting and outpatient setting. This was demonstrated in our case series since many patients go through multiple cycles of recovery and relapse but can still have improvement of their wounds with time. Furthermore, two of the four patients received dermal substitutes as treatment.

Given the barriers and stigma facing PWID, clinicians and health institutions must approach clinical care with nonjudgment and provide adequate medical care with a low-barrier and low-cost approach [10]. Another major problem that healthcare providers encounter when caring for PWID is patient-directed discharges and incomplete treatment. Currently, buprenorphine and methadone are considered first-line therapy for opiate withdrawal [12]. Ongoing research is exploring the use of short-acting opiate agonist therapy [13] and long-acting opiate agonist to increase retention in the hospital and decrease rates of incomplete treatment. When patients do decide to leave the hospital, particularly in patients presenting with severe wounds, we often provide wound care teaching, short-term wound care supplies, antibiotic prescriptions when needed, and instructions on when to return to seek care. In addition, counseling and resources related to addiction recovery should be provided with each encounter. As seen in our case series, it is not uncommon for patients to return to the hospital or get readmitted to continue medical care and wound care. PWID are typically subjected to discrimination even within the healthcare system, which further enhances mistrust leading to greater marginalization.

As challenges from fentanyl and xylazine continue to mount in the current opioid epidemic, healthcare systems must adapt and reimage care delivery to vulnerable populations. Innovative strategies must consider a harm-reduction approach to address these complex medical, social, and psychiatric needs.

## Data Availability

Data sharing is not applicable to this article as no new data were created or analyzed in this study.
